# Towards a Functional Understanding of Protein N-Terminal Acetylation

**DOI:** 10.1371/journal.pbio.1001074

**Published:** 2011-05-31

**Authors:** Thomas Arnesen

**Affiliations:** 1 Department of Molecular Biology, University of Bergen, Bergen, Norway; 2 Department of Surgery, Haukeland University Hospital, Bergen, Norway

## Abstract

Protein N-terminal acetylation is a major modification of eukaryotic proteins. Its functional implications include regulation of protein–protein interactions and targeting to membranes, as demonstrated by studies of a handful of proteins. Fifty years after its discovery, a potential general function of the N-terminal acetyl group carried by thousands of unique proteins remains enigmatic. However, recent functional data suggest roles for N-terminal acetylation as a degradation signal and as a determining factor for preventing protein targeting to the secretory pathway, thus highlighting N-terminal acetylation as a major determinant for the life and death of proteins. These contributions represent new and intriguing hypotheses that will guide the research in the years to come.

## N-Terminal Acetylation and N-Terminal Acetyltransferases

Chemical modifications of cellular proteins are a very common means of controlling their functions. The most commonly studied protein modification is phosphorylation, a key regulator of numerous proteins; however, eukaryotic proteins may undergo many different types of chemical modification, resulting in a plethora of protein variants within the cell. N-terminal acetylation (Nt-acetylation), which involves the transfer of an acetyl group from acetyl coenzyme A to the α-amino group of the first amino acid residue of a protein [1],[2], is among the most abundant of protein modifications. Despite being discovered over 50 years ago [3], we still do not understand the functional implications of Nt-acetylation for the thousands of proteins that are modified by it.

**Figure 1:**
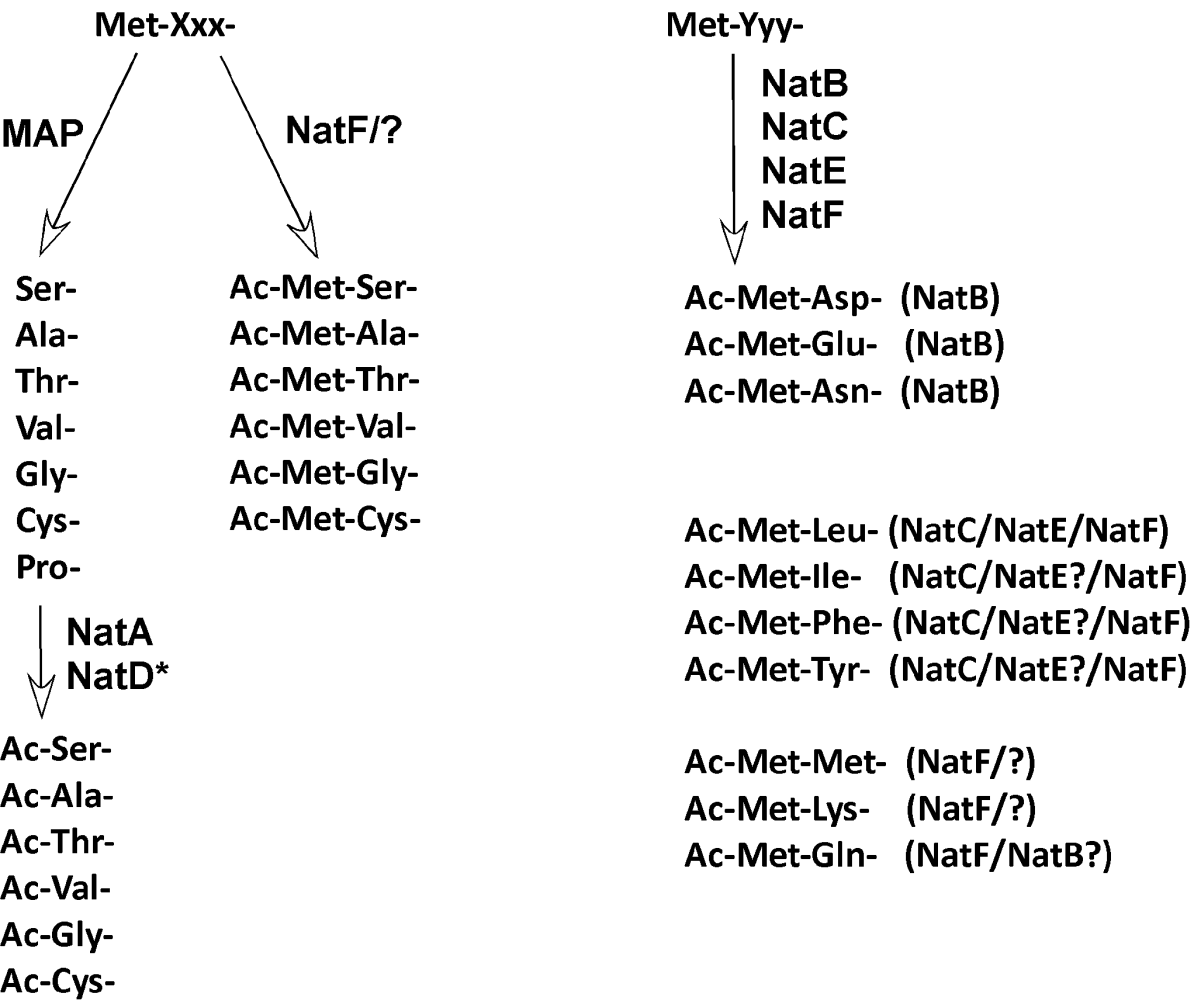
Schematic overview of N-terminal processing in eukaryotes. N-termini with small amino acid residues in the second position (Met-Xxx-) are mostly processed by methionine aminopeptidase (MAP), whereafter the newly generated N-termini may be acetylated by NatA (*or by NatD in the case of histones H2A and H4). This class of N-termini may also be acetylated on the initiator methionine (iMet) by unknown NATs or by NatF, which is specific for higher eukaryotes. N-termini with larger amino acid residues in the second position (Met-Yyy-) are not normally cleaved by MAPs, but potentially acetylated directly on the iMet by a variety of NATs depending on the N-terminal sequence. NatB potentially acetylates N-termini with acidic or hydrophilic residues in the second position. Hydrophobic N-termini are acetylated by NatC and potentially NatE, and in higher eukaryotes also NatF. NatF and perhaps other NATs acetylate Met-Met- and Met-Lys- N-termini. Information derived from [8] and references herein and NatF identification (P. Van Damme, K. Hole, A. Pimenta-Marques, J. Vandekerckhove, R. G. Martinho, et al., unpublished data).

**Figure 2:**
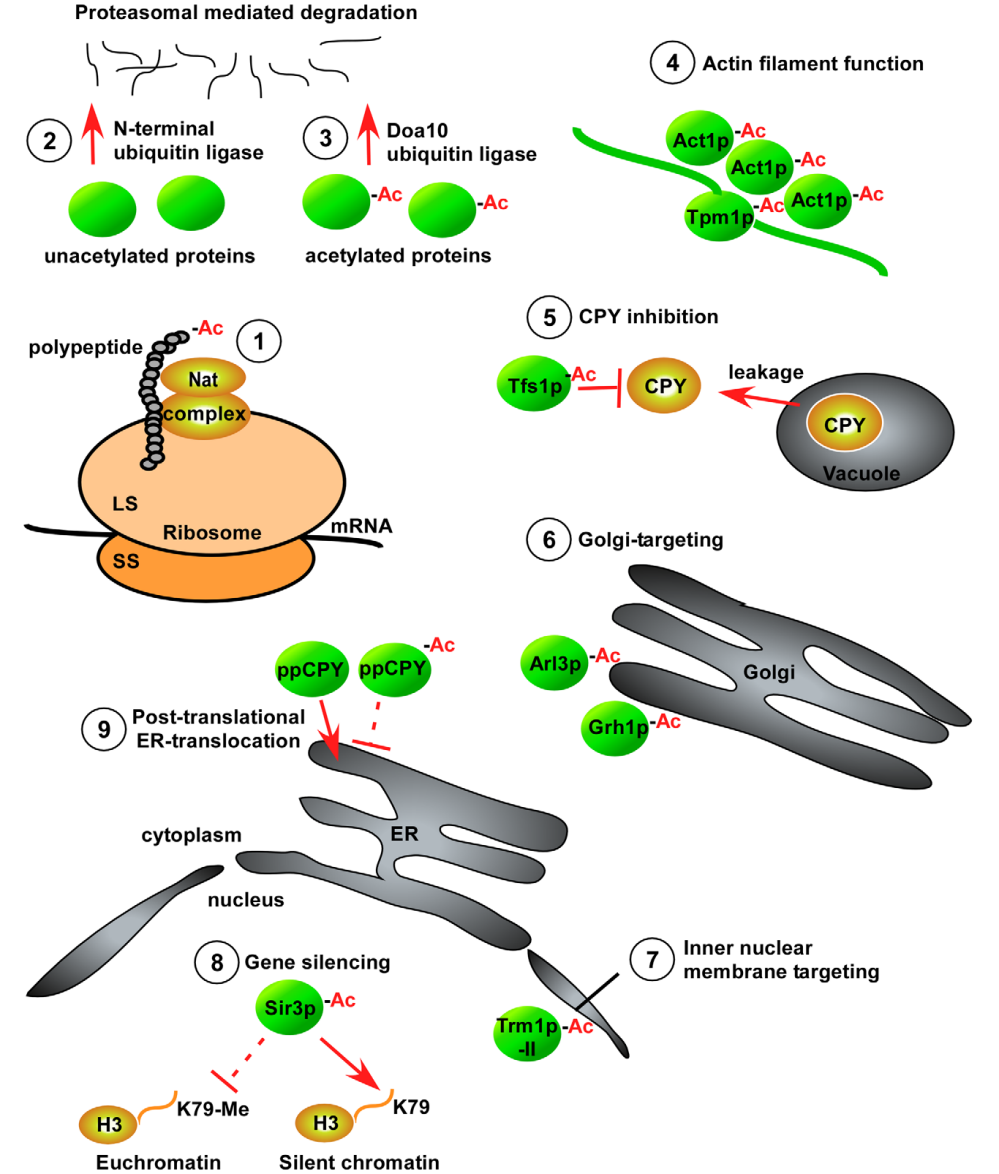
Functional effects of N-terminal acetylation. 1. Nat complexes associate with ribosomes to perform co-translational Nt-acetylation of a majority of eukaryotic proteins [8]. **2.** N-terminal ubiquitination promotes degradation of N-terminally unacetylated proteins, thus Nt-acetylation may protect proteins from this degradation pathway [15]. **3.** The newly discovered N-end rule branch involves the degradation of Ac-N-degrons via the Doa10 E3 ubiquitin ligase [18]. **4.** Nt-acetylation is essential for the functioning of actin filaments by modulating protein–protein interactions [21]–[23]. **5.** Tfs1 requires its acetylated N-terminus to directly inhibit the cytosolic carboxypeptidase CPY [24]. **6.** Nt-acetylation targets the GTPases Arl3p and Grh1p to the Golgi membrane [25]–[27]. **7.** Trm1p-II requires Nt-acetylation for proper association to the inner nuclear membrane [28]. **8.** Nt-acetylated Sir3p specifically interacts with unmethylated lysine 79 of histone H3 in silenced chromatin and is essential for proper gene silencing [32]. **9.** Nt-acetylation prevents post-translational translocation through the ER membrane [33].

Unlike most other protein modifications, Nt-acetylation is irreversible; it occurs mainly during the synthesis of the protein, catalyzed by N-terminal acetyltransferases (NATs) associated with ribosomes [4]–[7] (Figure 1 and Figure 2, point 1). There are several distinct NATs in eukaryotes—NatA‐NatF—each composed of one or more subunits and each acetylating a specific subgroup of N‐termini depending on the amino acid sequence of the first few amino acids [8]. The Nt‐acetylation patterns and the NAT machinery appear to be similar in all organisms from lower eukaryotes like the yeast *Saccharomyces cerevisiae* to higher eukaryotes [1], [9], [10], although higher eukaryotes have more protein Nt‐acetylation and express more NATs than yeast do [1], [8].

## N-Terminal Acetylation—Protein Stability, Degradation, and More

For many years, it was thought that Nt-acetylation protected proteins from degradation [11],[12]. Experimental data indeed indicated that proteins with acetylated N-termini were more stable in vivo than non-acetylated proteins [13]. One explanation for this might be the discovery in 2004 that another N-terminal modification—ubiquitination—involving direct attachment of the small protein ubiquitin to the N-terminal amino acid residue promotes the subsequent degradation of the protein [14]. Thus, blocking the N-terminus by Nt-acetylation potentially prevents N-terminal ubiquitination, and thus stabilizes the protein, as demonstrated, for instance, for p16 and p14/p19^ARF^ [14]–[16] (Figure 2, point 2). A naturally occurring N-terminally acetylated protein has not yet been found, however, that is N-terminally ubiquitinated and degraded when lacking its acetylation modification. An unacetylated N-terminus may still contribute to protein destabilization by a mechanism independent of ubiquitin [17].

In contrast to the general idea that Nt-acetylation protects proteins from degradation, recently Nt-acetylated amino acid sequences in certain proteins were found to be involved in creating degradation signals [18],[19]: a ubiquitin ligase, Doa10, recognizes Nt-acetylated proteins and marks them with ubiquitin for destruction (Figure 2, point 3). The study found this new class of degradation signal in eight yeast proteins, indicating that this is relevant to at least a subgroup of yeast proteins, and may potentially be a general phenomenon.

Determining which of these hypotheses (i.e., whether Nt-acetylation acts for or against protein stability) are correct vis-à-vis major protein populations will require proteome-scale investigations. Although these two hypotheses predict opposite functional outcomes for Nt-acetylation and thus appear to be contradictory, both mechanisms may take place side by side in the cell, each applying to specific subsets of proteins under defined conditions. A recent proteomics approach in yeast indicated that NatB-mediated acetylation did not generally affect protein stability, neither supporting N-terminal acetyl groups as stabilizers nor destabilizers [20]. Obviously, knowing that a majority of eukaryotic proteins are N-terminally acetylated, it would not make sense if these should all be automatically degraded due to their Nt-acetylation signals; however, cellular conditions might strongly influence the functional consequences of Nt-acetylation. If the Nt-acetylation signals are a part of a quality control mechanism to degrade unfolded or misfolded proteins and to regulate in vivo protein stoichiometries, as suggested by Varshavsky and co-workers, the degradation of specific proteins may vary greatly depending on cellular state [18]. Thus, more experiments representing the other major NATs as well as differential growth conditions and applied stresses are likely to reveal specific contributions of the N-terminal acetyl group for protein stability in vivo.

Other functional consequences of Nt-acetylation at the substrate level are confined to a handful of cases. The contractile proteins actin and tropomyosin have been shown to require NatB-mediated Nt-acetylation for proper function, specifically involving actin-tropomyosin binding and actomyosin regulation [21]–[23] (Figure 2, point 4). The lipid-binding protein Tfs1p also requires NatB-mediated acetylation to inhibit the carboxypeptidase Y (CPY), probably by a direct protein–protein interaction [24] (Figure 2, point 5). NatC-mediated acetylation was shown to target the GTPases Arl3p and Grh1p to the Golgi apparatus [25]–[27] (Figure 2, point 6), and acetylation is required for the association of the protein Trm1p-II with the inner nuclear membrane [28] (Figure 2, point 7). Although membrane targeting is a striking example of the functional importance of Nt-acetylation, this does not mean that Nt-acetylation is essential for protein localization in general, as demonstrated by the study of several NatB substrates where acetylation or a lack thereof had no apparent impact on subcellular localization [29]. NatA-mediated acetylation of Sir3p and Orc1p is essential for their role in gene silencing [30],[31]. More specifically, it was suggested that the acetylated Sir3p specifically interacts with lysine 79 of histone H3 in silenced chromatin whereas the unacetylated counterpart targeted also methylated H3K79 in euchromatin, thus reducing the specific binding to silenced regions [32] (Figure 2, point 8).

The data so far strongly suggest that Nt-acetylation plays a role in regulating protein stability and perhaps membrane targeting and gene silencing, although a general trend is not established. Clearly, even with recent seminal contributions, there is still a great need to understand the functional implications of Nt-acetylation at the proteome level. Obviously, there may be a variety of acetylation-dependent functions depending on the target protein, rather than one general function. There is even the possibility that this modification affects the function of only very few proteins.

## N-Terminal Acetylation and Endoplasmic Reticulum Translocation

In this issue of *PLoS Biology*, Forte, Pool, and Stirling present an interesting hypothesis linking the lack of Nt-acetylation to the ability of a protein to be translocated through the endoplasmic reticulum (ER) and into the secretory pathway [33]. In silico analyses correlating the N-terminal processing status (i.e., N-terminal methionine cleavage and Nt-acetylation) and the presence of signal peptides (which target proteins to the ER) revealed a strong correlation between being unprocessed and being translocated. Functional studies altering a normally secreted protein from an unacetylated to an acetylated state also inhibited translocation, suggesting that Nt-acetylation may be necessary for cytosolic retention (Figure 2, point 9). Importantly, the inhibitory effect on translocation of certain residues at position 2 depends upon the relevant N-terminal processing machinery [33].

Two major mechanisms move secretory and membrane proteins from the cytosol through the Sec61 translocon channel and into the lumen of the ER. The first is signal recognition particle (SRP)-dependent co-translational translocation; the second also involves post-translational translocation and is Sec62 dependent. Which pathway a protein enters depends on the hydrophobicity of the central core of its 15–30 residue long N-terminal targeting sequence [34],[35]. In the case of co-translational translocation, the signal sequences with the most hydrophobic cores are recognized by SRP, which targets the ribosome nascent chain (RNC) complex to the Sec61 translocon via the SRP receptor (SR). The ribosome and the translocon bind tightly and the nascent polypeptide is allowed to enter the translocation channel [36]–[39]. Post-translational translocation occurs after the protein has been fully made. Cytosolic chaperones maintain the polypeptide in a state that is compatible with subsequent translocation. These proteins are also transported through the Sec61 translocon, but requires rather binding to the Sec62 complex, while in this case SRP and SR are not involved [35],[40]–[43].

Interestingly, the proteins Forte et al. found retained in the cytosol when acetylated all depended on Sec62. The Nt-acetylated protein was not properly targeted to the Sec61 translocon, meaning that the acetyl group most likely disrupts the interaction with either the translocon itself or one of the initial targeting factors (i.e., chaperones or the Sec62 complex). A co-translationally SRP-dependent translocated protein was not affected even when having a sequence that would normally lead to Nt-acetylation. In fact, the acetylation-prone sequence did not result in acetylation of the given N-terminus, thus it appears like the binding of SRP precedes and prevents any potential further processing by NATs (and perhaps also Methionine aminopeptidases). This is expected given that in eukaryotes, the signal sequence of a transmembrane protein may facilitate the binding between RNC and SRP even before the signal sequence emerges from the ribosomal tunnel, thus restricting the availability for processing enzymes [44]. However, the absoluteness in SRP dominance over processing enzymes awaits more comprehensive investigations. Further, in the case of co-translational SRP-dependent translocation, we do not know whether Nt-acetylation would, if present, cause defective translocation or not. However, this question will most likely remain hypothetical since the processing enzymes probably will be kept at a distance once the SRP has engaged. Since several proteins can utilize both the co- and the post-translational pathways, avoiding acetylation at the N-terminus would still be a prerequisite for proper translocation.

N-terminal signal sequences in yeast proteins often had lysine or arginine in the second position which in most cases are predicted to have no Nt-acetylation [33]. These residues are also abundant in human signal sequences, although not to such a great extent, potentially reflecting the fact that the Nt-acetylation machinery in higher eukaryotes, but not yeast, includes NatF, which acetylates protein N-termini with lysine in the second position (Figure 1) (P. Van Damme, K. Hole, A. Pimenta-Marques, J. Vandekerckhove, R. G. Martinho, et al., submitted). In that light it will be interesting to see if this rule applies to human proteins as well, and whether the signal sequences have adapted to the presence of an extended acetylation capacity in higher eukaryotes. One may also speculate whether the acetylation machinery in higher eukaryotes might have evolved to facilitate evolutionary changes in the translocation processes, for instance to ensure cytosolic localization for proteins otherwise destined for translocation. Experimental analyses of signal sequences of higher eukaryotes and their acetylation status will hopefully enlighten us in the years to come. Although the study by Forte, Pool, and Stirling clearly shows that yeast proteins need to be unacetylated in order to get translocated post-translationally, we do not yet know whether any naturally occurring acetylated cytosolic proteins would actually get translocated if they were not Nt-acetylated, meaning that acetylation would represent an extra layer of stringency in order to ensure that proteins destined to live in the cytosol actually reside in the cytosol.

## What Next?

Some of the challenge in understanding the functional implications of Nt-acetylation lies in the fact that this modification is considered irreversible. If a protein is Nt-acetylated at birth, it will probably remain that way until its death. This means that it is difficult to envision its involvement in highly regulatory pathways that require an on/off switch or a rapid functional modulation. However, given that the majority of eukaryotic proteins carry this modification it seems highly unlikely that this is functionally relevant only for the few cases where a function has been demonstrated this far (Figure 2). To this end, the recent hypotheses involving Nt-acetylation in mediating degradation [18] and prevention of translocation [33] may in fact represent major clues to why this modification has evolved. So far, the evidence at hand is solid and it is very likely that these two functional links are important in eukaryotes. However, the overall understanding of how these phenomena come to play in vivo is not yet revealed. Proteome-wide analyses, assessing the generality and the rules applying, should be carried out. A genetic model like *S. cerevisiae* where specific NATs have been deleted, combined with proteomics as well as functional translocation studies, might be one way to address this at the endogenous substrate level. Also, testing specific endogenous substrates by removing their specific acetylation by the XPX-rule [9] (having a proline at the second position will prevent Nt-acetylation) using, for instance, yeast or fruit fly models, would be productive. Alternatively, introducing Nt-acetylation-prone N-termini to a large number of unacetylated proteins destined for different translocation routes would speak for the generality of the hypothesis. Furthermore, it is essential to get a detailed mechanistic understanding of the processes. For instance, why is a protein with an Nt-acetylated signal sequence not capable of being post-translationally translocated? Will the acetyl group steer the nascent chain towards an interaction with the chaperones specialized for cytosolic proteins rather than the set of chaperones required for targeting to the translocon? In order for post-translational translocation to occur, proteins need to stay in an unfolded state. Thus, if the acetyl group acts as the first seed promoting folding, this may determine whether translocation will occur or not. Once acetylated, and thus retained in the cytosol, the protein will be susceptible to the Ac-N-degron-mediated destruction. As such, the cell might first steer protein targeting via Nt-acetylation, after which the Ac-N-degron fine tunes cytosolic protein levels and gets rid of misfolded and unfolded proteins.

Far from being an inert, common, and annoying modification (because it interferes with protein sequencing methods), Nt-acetylation now emerges as a major determinant for the life and death of proteins. Without question, much is determined from birth. That goes for proteins as well.

## Abbreviations

ER: endoplasmic reticulum
MAP: methionine aminopeptidase
NAT: N-terminal acetyltransferase
Nt-acetylation: N-terminal acetylation
RNC: ribosome nascent chain
SR: signal recognition particle receptor
SRP: signal recognition particle
